# *N*,*N*-Bis(4-chloro­phenyl­sulfon­yl)succinamide dihydrate

**DOI:** 10.1107/S1600536812024725

**Published:** 2012-06-13

**Authors:** H. Purandara, Sabine Foro, B. Thimme Gowda

**Affiliations:** a Department of Chemistry, Mangalore University, Mangalagangotri 574 199, Mangalore, India; b Institute of Materials Science, Darmstadt University of Technology, Petersenstrasse 23, D-64287 Darmstadt, Germany

## Abstract

The asymmetric unit of the title compound, C_16_H_14_Cl_2_N_2_O_6_S_2_·2H_2_O, contains one half-mol­ecule of *N*,*N*-bis­(4-chloro­phenyl­sulfon­yl)succinamide, with a centre of symmetry at the mid-point of the central C—C bond, and one water mol­ecule. The succinamide mol­ecules are not directly connected *via* hydrogen bonds, but by hydrogen bonds *via* the water mol­ecules.

## Related literature

For our studies on the effects of substituents on the structures and other aspects of *N*-(ar­yl)-amides, see: Gowda *et al.* (2000 ▶); Rodrigues *et al.* (2011 ▶), of *N*-chloro­aryl­amides, see: Gowda & Rao (1989 ▶); Jyothi & Gowda (2004 ▶) and of *N*-bromo­aryl­sulfonamides, see: Gowda & Mahadevappa (1983 ▶); Usha & Gowda (2006 ▶).
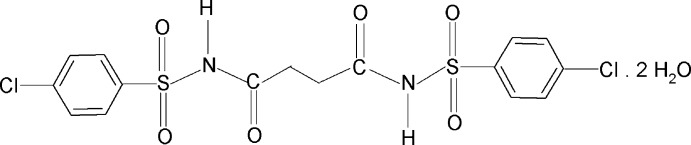


## Experimental

### 

#### Crystal data

C_16_H_14_Cl_2_N_2_O_6_S_2_·2H_2_O
*M* *_r_* = 501.34
Monoclinic, 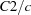

*a* = 33.349 (2) Å
*b* = 4.9737 (4) Å
*c* = 13.171 (1) Å
β = 90.660 (7)°
*V* = 2184.5 (3) Å^3^
*Z* = 4
Mo *K*α radiation
μ = 0.53 mm^−1^
*T* = 293 K
0.48 × 0.40 × 0.12 mm

#### Data collection

Oxford Diffraction Xcalibur diffractometer with a Sapphire CCD detector
Absorption correction: multi-scan (*CrysAlis RED*; Oxford Diffraction, 2009 ▶) *T*_min_ = 0.784, *T*_max_ = 0.939
3837 measured reflections
2233 independent reflections
1906 reflections with *I* > 2σ(*I*)
*R*_int_ = 0.012

#### Refinement

*R*[*F* ^2^ > 2σ(*F* ^2^)] = 0.046
*wR*(*F* ^2^) = 0.120
*S* = 1.06
2233 reflections
139 parameters
7 restraints
H atoms treated by a mixture of independent and constrained refinement
Δρ_max_ = 0.55 e Å^−3^
Δρ_min_ = −0.49 e Å^−3^

### 

Data collection: *CrysAlis CCD* (Oxford Diffraction, 2009 ▶); cell refinement: *CrysAlis CCD*; data reduction: *CrysAlis RED* (Oxford Diffraction, 2009 ▶); program(s) used to solve structure: *SHELXS97* (Sheldrick, 2008 ▶); program(s) used to refine structure: *SHELXL97* (Sheldrick, 2008 ▶); molecular graphics: *PLATON* (Spek, 2009 ▶); software used to prepare material for publication: *SHELXL97*.

## Supplementary Material

Crystal structure: contains datablock(s) I, global. DOI: 10.1107/S1600536812024725/bt5934sup1.cif

Structure factors: contains datablock(s) I. DOI: 10.1107/S1600536812024725/bt5934Isup2.hkl

Supplementary material file. DOI: 10.1107/S1600536812024725/bt5934Isup3.cml

Additional supplementary materials:  crystallographic information; 3D view; checkCIF report

## Figures and Tables

**Table 1:** Hydrogen-bond geometry (Å, °)

*D*—H⋯*A*	*D*—H	H⋯*A*	*D*⋯*A*	*D*—H⋯*A*
N1—H1*N*⋯O4^i^	0.84 (2)	1.90 (2)	2.735 (3)	178 (3)
O4—H41⋯O3	0.87	2.27	3.137 (4)	177
O4—H42⋯O2^ii^	0.84	2.23	2.941 (3)	142
O4—H42⋯O3^ii^	0.84	2.58	3.262 (4)	139

Symmetry codes: (i) 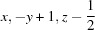; (ii) 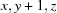.

**Fig. 1.**
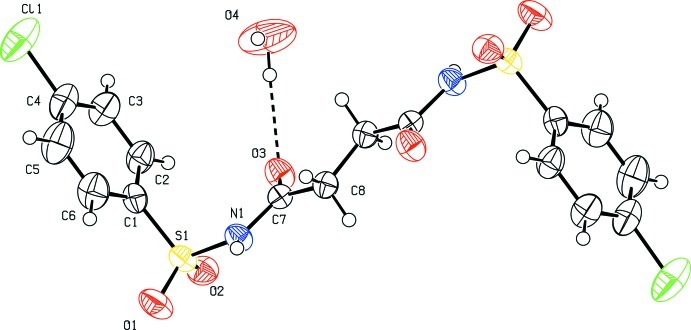
Molecular structure of the title compound, showing the atom labelling scheme with the displacement ellipsoids drawn at the 50% probability level.

**Fig. 2.**
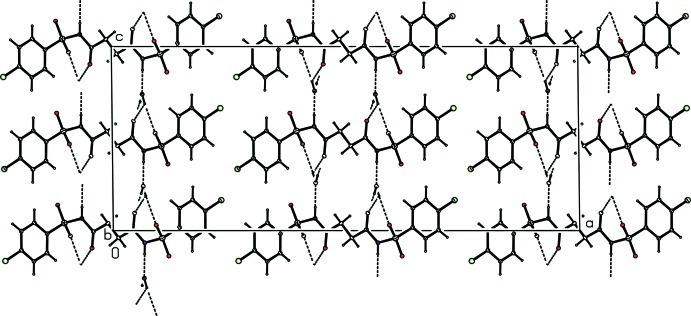
Molecular packing of the title compound with hydrogen bonding shown as dashed lines.

## supplementary crystallographic information

### Comment

As part of our studies on the substituent effects on the structures and other aspects of *N*-(aryl)-amides (Gowda *et al.*, 2000; Rodrigues *et al.*, 2011); *N*-chloroarylsulfonamides (Gowda & Rao, 1989; Jyothi & Gowda, 2004) and *N*-bromoaryl- sulfonamides (Gowda & Mahadevappa, 1983; Usha & Gowda, 2006), in the present work, the crystal structure of *N*,*N*-bis(4-chlorophenylsulfonyl)succinamide dihydrate has been determined (Fig. 1).

In the two C—SO_2_—NH—CO—CH_2_ central segments of the structure, the N—H, C=O and C—H bonds are *anti* to the adjacent bonds, similar to that observed in *N*,*N*-bis(4-chlorophenylsulfonyl)-adipamide (I) (Rodrigues *et al.*, 2011). The orientations of sulfonamide groups with respect to the attached phenyl rings are given by the torsion angles of C2—C1—S1—N1 = 91.0 (2)° and C6—C1—S1—N1 = -87.6 (2)°. The molecule is bent at the S atom with the C1—S1—N1—C7 torsion angle of -70.0 (2)°, compared to the values of 55.0 (6)° in (I).

The dihedral angle between the benzene ring and the SO_2_—NH—C(O)—C segment in the two halves of the molecule is 78.0 (2)°, compared to the value of 83.5 (2)° in (I).

One of the water H-atoms exhibits bifurcated H-bonding with one of the O-atoms of SO_2_ group and the amide O-atom.

A series of N—H···O(W) and O–H···O(S and C) intermolecular hydrogen bonds (Table 1) link the molecules into infinite chains running along *c*-axis (Fig. 2).

### Experimental

Succinic anhydride (0.015 mole), *N*,*N*'-dicyclohexylcarbodiimide (0.01mole) and 4-dimethylaminopyridine (0.004 mole) were added to a solution of 4-chlorobenzenesulfonamide (0.01 mole) in dichloromethane. The mixture was strirred for 24 h at room temperature and set aside for completion of the reaction.. The reaction mixture was filtered to remove the by-product *N*,*N*'-dicyclohexylurea. The filtrate was diluted with water and then the organic layer was extracted. The latter was washed with water to remove the base and the succinic anhydride, dried over anhydrous sodium sulfate and filtered. The filtrate was concentrated to dryness. The residue was recrystallized to constant melting point from ethyl acetate (163–165 °C). The purity of the compound was checked and characterized by its infrared spectrum.

Plate like colorless single crystals used in X-ray diffraction studies were grown in ethyl acetate solution by slow evaporation of the solvent from its solution at room temperature.

### Refinement

The H atoms of the water molecule were located in difference map and were refined as riding on their parent O atom. H atoms bonded to C were positioned with idealized geometry using a riding model with the aromatic C—H = 0.93 Å, methylene C—H = 0.97 Å. All H atoms were refined with their isotropic displacement parameter set to 1.2 times of the *U*_eq_ of the parent atom. The amino H atom was freely refined with the N-H distance restrained to 0.86 (2)Å. The displacement ellipsoid of the water O atom was restrained to an isotropic bahaviour.

### Crystal data

**Table:**

C_16_H_14_Cl_2_N_2_O_6_S_2_·2H_2_O	*F*(000) = 1032
*M**_r_* = 501.34	*D*_x_ = 1.524 Mg m^−^^3^
Monoclinic, *C*2/*c*	Mo *K*α radiation, λ = 0.71073 Å
Hall symbol: -C 2yc	Cell parameters from 1880 reflections
*a* = 33.349 (2) Å	θ = 2.9–27.9°
*b* = 4.9737 (4) Å	µ = 0.53 mm^−^^1^
*c* = 13.171 (1) Å	*T* = 293 K
β = 90.660 (7)°	Plate, colourless
*V* = 2184.5 (3) Å^3^	0.48 × 0.40 × 0.12 mm
*Z* = 4	

### Data collection

**Table:**

Oxford Diffraction Xcalibur diffractometer with a Sapphire CCD detector	2233 independent reflections
Radiation source: fine-focus sealed tube	1906 reflections with *I* > 2σ(*I*)
Graphite monochromator	*R*_int_ = 0.012
Rotation method data acquisition using ω and phi scans	θ_max_ = 26.4°, θ_min_ = 3.1°
Absorption correction: multi-scan (*CrysAlis RED*; Oxford Diffraction, 2009)	*h* = −31→41
*T*_min_ = 0.784, *T*_max_ = 0.939	*k* = −6→2
3837 measured reflections	*l* = −16→15

### Refinement

**Table:**

Refinement on *F*^2^	Primary atom site location: structure-invariant direct methods
Least-squares matrix: full	Secondary atom site location: difference Fourier map
*R*[*F*^2^ > 2σ(*F*^2^)] = 0.046	Hydrogen site location: inferred from neighbouring sites
*wR*(*F*^2^) = 0.120	H atoms treated by a mixture of independent and constrained refinement
*S* = 1.06	*w* = 1/[σ^2^(*F*_o_^2^) + (0.0516*P*)^2^ + 3.9644*P*] where *P* = (*F*_o_^2^ + 2*F*_c_^2^)/3
2233 reflections	(Δ/σ)_max_ = 0.004
139 parameters	Δρ_max_ = 0.55 e Å^−^^3^
7 restraints	Δρ_min_ = −0.49 e Å^−^^3^

### Special details

**Table:**

Experimental. Absorption correction: CrysAlis RED (Oxford Diffraction, 2009) Empirical absorption correction using spherical harmonics, implemented in SCALE3 ABSPACK scaling algorithm.
Geometry. All e.s.d.'s (except the e.s.d. in the dihedral angle between two l.s. planes) are estimated using the full covariance matrix. The cell e.s.d.'s are taken into account individually in the estimation of e.s.d.'s in distances, angles and torsion angles; correlations between e.s.d.'s in cell parameters are only used when they are defined by crystal symmetry. An approximate (isotropic) treatment of cell e.s.d.'s is used for estimating e.s.d.'s involving l.s. planes.
Refinement. Refinement of *F*^2^ against ALL reflections. The weighted *R*-factor *wR* and goodness of fit *S* are based on *F*^2^, conventional *R*-factors *R* are based on *F*, with *F* set to zero for negative *F*^2^. The threshold expression of *F*^2^ > σ(*F*^2^) is used only for calculating *R*-factors(gt) *etc*. and is not relevant to the choice of reflections for refinement. *R*-factors based on *F*^2^ are statistically about twice as large as those based on *F*, and *R*- factors based on ALL data will be even larger.

### Fractional atomic coordinates and isotropic or equivalent isotropic displacement parameters (Å^2^)

**Table:**

	*x*	*y*	*z*	*U*_iso_*/*U*_eq_	
Cl1	0.23218 (3)	0.6607 (2)	0.16525 (9)	0.0914 (4)	
S1	0.104840 (19)	−0.08075 (14)	−0.04102 (5)	0.0398 (2)	
O1	0.11860 (7)	−0.1623 (5)	−0.13839 (15)	0.0590 (6)	
O2	0.09204 (6)	−0.2788 (4)	0.02984 (15)	0.0487 (5)	
O3	0.04604 (6)	0.1896 (4)	0.09543 (13)	0.0474 (5)	
N1	0.06718 (7)	0.1229 (5)	−0.06616 (16)	0.0398 (5)	
H1N	0.0665 (9)	0.182 (6)	−0.1257 (16)	0.048*	
C1	0.14188 (7)	0.1197 (5)	0.01713 (19)	0.0388 (6)	
C2	0.14276 (8)	0.1472 (6)	0.1219 (2)	0.0480 (7)	
H2	0.1245	0.0541	0.1616	0.058*	
C3	0.17086 (9)	0.3135 (7)	0.1669 (2)	0.0558 (8)	
H3	0.1716	0.3346	0.2370	0.067*	
C4	0.19760 (9)	0.4469 (7)	0.1072 (3)	0.0565 (8)	
C5	0.19727 (10)	0.4182 (8)	0.0030 (3)	0.0700 (10)	
H5	0.2158	0.5097	−0.0363	0.084*	
C6	0.16932 (10)	0.2536 (7)	−0.0421 (2)	0.0584 (8)	
H6	0.1688	0.2322	−0.1123	0.070*	
C7	0.04271 (7)	0.2394 (5)	0.00549 (19)	0.0356 (5)	
C8	0.01137 (7)	0.4226 (6)	−0.03950 (19)	0.0401 (6)	
H81	−0.0074	0.3163	−0.0794	0.048*	
H82	0.0242	0.5487	−0.0850	0.048*	
O4	0.06513 (12)	0.6735 (8)	0.2409 (2)	0.1206 (13)	
H41	0.0606	0.5373	0.2014	0.145*	
H42	0.0704	0.7633	0.1885	0.145*	

### Atomic displacement parameters (Å^2^)

**Table:**

	*U*^11^	*U*^22^	*U*^33^	*U*^12^	*U*^13^	*U*^23^
Cl1	0.0731 (6)	0.0863 (7)	0.1142 (9)	−0.0383 (6)	−0.0254 (6)	0.0188 (6)
S1	0.0432 (4)	0.0384 (4)	0.0379 (3)	0.0079 (3)	0.0027 (2)	−0.0028 (3)
O1	0.0674 (13)	0.0645 (14)	0.0451 (11)	0.0189 (11)	0.0070 (9)	−0.0130 (10)
O2	0.0542 (12)	0.0363 (10)	0.0554 (12)	−0.0005 (9)	−0.0006 (9)	0.0030 (9)
O3	0.0513 (11)	0.0546 (12)	0.0365 (10)	0.0092 (9)	0.0058 (8)	0.0037 (9)
N1	0.0432 (12)	0.0440 (13)	0.0322 (10)	0.0089 (10)	−0.0001 (9)	−0.0004 (9)
C1	0.0340 (12)	0.0399 (14)	0.0425 (13)	0.0055 (11)	0.0039 (10)	0.0063 (11)
C2	0.0418 (14)	0.0598 (18)	0.0424 (14)	−0.0101 (13)	0.0040 (11)	0.0073 (13)
C3	0.0507 (16)	0.067 (2)	0.0496 (16)	−0.0113 (15)	−0.0025 (13)	0.0042 (15)
C4	0.0418 (15)	0.0547 (18)	0.073 (2)	−0.0087 (14)	−0.0095 (14)	0.0147 (16)
C5	0.0564 (19)	0.081 (3)	0.073 (2)	−0.0213 (18)	0.0103 (16)	0.025 (2)
C6	0.0558 (17)	0.073 (2)	0.0469 (16)	−0.0075 (16)	0.0102 (13)	0.0139 (15)
C7	0.0332 (12)	0.0350 (13)	0.0388 (13)	−0.0021 (10)	0.0010 (10)	−0.0040 (10)
C8	0.0348 (12)	0.0436 (14)	0.0419 (13)	0.0021 (11)	−0.0027 (10)	−0.0035 (12)
O4	0.180 (3)	0.131 (3)	0.0514 (14)	−0.070 (2)	0.0235 (17)	−0.0267 (17)

### Geometric parameters (Å, º)

**Table:**

Cl1—C4	1.739 (3)	C3—C4	1.367 (4)
S1—O2	1.426 (2)	C3—H3	0.9300
S1—O1	1.426 (2)	C4—C5	1.380 (5)
S1—N1	1.644 (2)	C5—C6	1.371 (5)
S1—C1	1.756 (3)	C5—H5	0.9300
O3—C7	1.214 (3)	C6—H6	0.9300
N1—C7	1.382 (3)	C7—C8	1.503 (3)
N1—H1N	0.837 (19)	C8—C8^i^	1.506 (5)
C1—C6	1.381 (4)	C8—H81	0.9700
C1—C2	1.387 (4)	C8—H82	0.9700
C2—C3	1.379 (4)	O4—H41	0.8663
C2—H2	0.9300	O4—H42	0.8435
			
O2—S1—O1	119.62 (13)	C3—C4—Cl1	118.5 (3)
O2—S1—N1	108.89 (12)	C5—C4—Cl1	119.9 (2)
O1—S1—N1	104.31 (12)	C6—C5—C4	119.4 (3)
O2—S1—C1	108.73 (12)	C6—C5—H5	120.3
O1—S1—C1	108.76 (13)	C4—C5—H5	120.3
N1—S1—C1	105.65 (12)	C5—C6—C1	119.7 (3)
C7—N1—S1	125.20 (18)	C5—C6—H6	120.2
C7—N1—H1N	119 (2)	C1—C6—H6	120.2
S1—N1—H1N	114 (2)	O3—C7—N1	122.2 (2)
C6—C1—C2	120.6 (3)	O3—C7—C8	124.3 (2)
C6—C1—S1	119.6 (2)	N1—C7—C8	113.5 (2)
C2—C1—S1	119.8 (2)	C7—C8—C8^i^	113.0 (3)
C3—C2—C1	119.5 (3)	C7—C8—H81	109.0
C3—C2—H2	120.2	C8^i^—C8—H81	109.0
C1—C2—H2	120.2	C7—C8—H82	109.0
C4—C3—C2	119.3 (3)	C8^i^—C8—H82	109.0
C4—C3—H3	120.4	H81—C8—H82	107.8
C2—C3—H3	120.4	H41—O4—H42	87.6
C3—C4—C5	121.6 (3)		
			
O2—S1—N1—C7	46.9 (3)	C2—C3—C4—C5	−0.3 (5)
O1—S1—N1—C7	175.7 (2)	C2—C3—C4—Cl1	178.8 (3)
C1—S1—N1—C7	−69.7 (2)	C3—C4—C5—C6	0.5 (6)
O2—S1—C1—C6	155.6 (2)	Cl1—C4—C5—C6	−178.6 (3)
O1—S1—C1—C6	23.9 (3)	C4—C5—C6—C1	0.2 (6)
N1—S1—C1—C6	−87.6 (3)	C2—C1—C6—C5	−1.0 (5)
O2—S1—C1—C2	−25.8 (3)	S1—C1—C6—C5	177.5 (3)
O1—S1—C1—C2	−157.6 (2)	S1—N1—C7—O3	−3.5 (4)
N1—S1—C1—C2	90.9 (2)	S1—N1—C7—C8	178.49 (19)
C6—C1—C2—C3	1.1 (5)	O3—C7—C8—C8^i^	9.9 (4)
S1—C1—C2—C3	−177.4 (2)	N1—C7—C8—C8^i^	−172.1 (3)
C1—C2—C3—C4	−0.5 (5)		

Symmetry code: (i) −*x*, −*y*+1, −*z*.

### Hydrogen-bond geometry (Å, º)

**Table:**

*D*—H···*A*	*D*—H	H···*A*	*D*···*A*	*D*—H···*A*
N1—H1*N*···O4^ii^	0.84 (2)	1.90 (2)	2.735 (3)	178 (3)
O4—H41···O3	0.87	2.27	3.137 (4)	177
O4—H42···O2^iii^	0.84	2.23	2.941 (3)	142
O4—H42···O3^iii^	0.84	2.58	3.262 (4)	139

Symmetry codes: (ii) *x*, −*y*+1, *z*−1/2; (iii) *x*, *y*+1, *z*.
